# Surgical techniques and function outcome for cingulate gyrus glioma, how we do it

**DOI:** 10.3389/fonc.2022.986387

**Published:** 2022-09-26

**Authors:** Fangyuan Gong, Lei Jin, Qiuwei Song, Zhong Yang, Hong Chen, Jinsong Wu

**Affiliations:** ^1^ Department of Neurosurgery, Huashan Hospital, Fudan University, Shanghai, China; ^2^ Department of Nursing, Huashan Hospital, Fudan University, Shanghai, China; ^3^ Department of Radiology, Huashan Hospital, Fudan University, Shanghai, China; ^4^ Department of Pathology, Huashan Hospital, Fudan University, Shanghai, China

**Keywords:** cingulate, glioma, subregion, surgery, prognosis, oncology

## Abstract

**Objective:**

Cingulate cortex and cingulum both play crucial roles in limbic system. The aim of study is to observe and analyze surgical outcomes of cingulate gyrus glioma through extents of resection (EORs), overall survival (OS), and postsurgical neurological outcome.

**Method:**

The authors retrospectively studied 95 consecutive adult cases of primary cingulate gliomas that all underwent craniotomies and tumor resection. The patients were classified into unitary sub-region based on the four-division model. The information of clinical symptoms, pathology, EOR, postoperative neurological outcome and survival were analyzed through group comparison.

**Result:**

Low-grade gliomas (LGGs) were more prevalent (69.47%) for cingulate gyrus. Diffuse astrocytoma (40.00%) was most common histopathological diagnosis in total. Regarding sub-regions tumor involved in, midcingulate cortex (MCC) glioma was most prevalent (54.74%) followed by anterior cingulate cortex (ACC) glioma. Among all patients, 83 patients (87.37%) received EOR ≥ 90%. In LGG group, 58 patients (87.88%) received EOR ≥ 90%. The achievement of EOR significantly correlated with survival (P = 0.006). MCC cases were significantly associated with short-term morbidity in either language or motor function (P = 0.02). Majority of ACC cases (80.65%) escaped from any short-term deficits and nearly 90% free for permanent morbidity. Tumors in the dominant hemisphere were significantly associated with language dysfunction or cognition dysfunction, either short-term (P=0.0006) or long-term morbidity (P=0.0111). Age was the only postoperative susceptible predictor for all types of transient (P=0.021) and permanent (P=0.02) neurological deficit.

**Conclusion:**

Regarding cingulate gyrus glioma, the management of surgical plans could be carried out into four sub-region level. In spite of short-term neurological dysfunction caused by surgical procedure, majority of transient dysfunction could be relieved or recovered in long-term. The necessary effort to prolong overall survival is still to achieve advisable EOR.

## Introduction

The cingulate gyrus glioma is found on the medial side of the hemisphere side where it extends from the lamina terminalis to the retrosplenial cortex between the cingulate sulcus and the corpus callosum sulcus. The cingulate gyrus belongs to the mesocortex, also called the juxtallocortex. It is formed at borders between the isocortex and allocortex-like transitional areas of the cerebral cortex (1). Regarding Brodmann’s anatomy, the cingulate gyrus mainly involves area 24 on the anterior side, and area 23 on the posterior side. Areas 29 and 30, such as the retrosplenial cortex, are also involved in the posterior depths of the callosal sulcus (2). In the past neuroanatomists and radiologists had demonstrated that the cingulate gyrus plays a considerable role in the Papez circuit which was close to emotion function (3, 4). Recently, more studies provided evidence that the cingulate gyrus is related to motor control and cognition formation, especially memory (5, 6). A division of the cingulate gyrus, the four-region model, is proposed based on cytoarchitectural and functional studies (7). Vogt systematically summarized the locations, borders, sub-regions, and main functions of each sub-region. Based on the four-subregion model, it is feasible to observe particular surgical outcomes for clinical practice, like the association between supplementary motor area syndrome and cingulate dissection (8).

Glioma is the most common malignant tumor of the central nervous system, but cerebral tumors that derive from the cingulate gyrus are comparatively rare among supratentorial gliomas. A study reported that only 3.5% of all surgically treated supratentorial gliomas in the department are in the anterior or posterior cingulate gyrus (9). Yet, a few types of cingulate glioma are spread to the surrounding supra-cingular cortex. Previous gross anatomy and functional imaging studies revealed the presence of underlying subcortical fibers that connect ACC with frontal cortex or PCC with parietal cortex (10, 11). Meanwhile MCC is a region that projects to the spinal cord and regulates skeletomotor function (12). Some researchers found that the cingulate motor cortex is associated with facial expression based on observations following cortical stimulation, functional neuroimaging, and localized surgical resection (13). Therefore previous neurosurgeons described multiple surgical deficits after cingulotomies such as motor deficits (14) and even attentional deficits (15). However, the extent of resection that should be carried out on cingulate tumor resection despite function deficits, is still unknown. The four-subregion model not only enables neurosurgeons to elaborately arrange surgical plans, but it also provides insights on surgical outcomes.

In this study, we retrospectively observed a series of 95 cingulate gyrus glioma patients with a focus on the different sub-regions of the gliomas after craniotomies. We analyzed assessing results of function outcomes using a cognition test, muscle strength measurement, and language battery. Through short-term and long-term follow-ups, the manifestations of function morbidity and overall survival were reported. The purpose of this study is to identify associating factors for functional outcome and survival, while also reviewing and summarizing surgical techniques and perioperative management based on the sub-region model.

## Method

### Patient selection and image acquisition

In this study, we retrospectively enrolled 95 consecutive adult cases of cingulate gyrus (CG) glioma from 2015 to 2021. All patients were pathologically diagnosed with WHO grades 2 to 4 primary gliomas. For each patient, the tumor histopathology was reviewed by a senior pathologist according to the 5th Edition of WHO Classification of Central Nervous System Tumours (WHO CNS5). The following clinical characteristics of patients were recorded: age at diagnosis, gender, symptoms for main chief complaints, tumor laterality, WHO grade, histopathology, molecular pathological markers (IDH and chromosome 1p/19q), and postoperative adjuvant therapy. All patients performed craniotomy surgeries in Huashan Hospital, Shanghai. All surgeries were planned and performed by senior neurosurgeons. One to three days before the operation, the patient would take high-resolution structural MRI (3.0T, MAGNETOM Verio, Siemens®, Germany) examination to reconstruct 3-dimension images which were used to judge the preoperative boundary (FLAIR or T2-weighted sequence for LGGs and contrast-enhancing tissue on gadolinium-contrasted T1-weighted images for HGGs).

### Categories and classification

**Figure 1:**
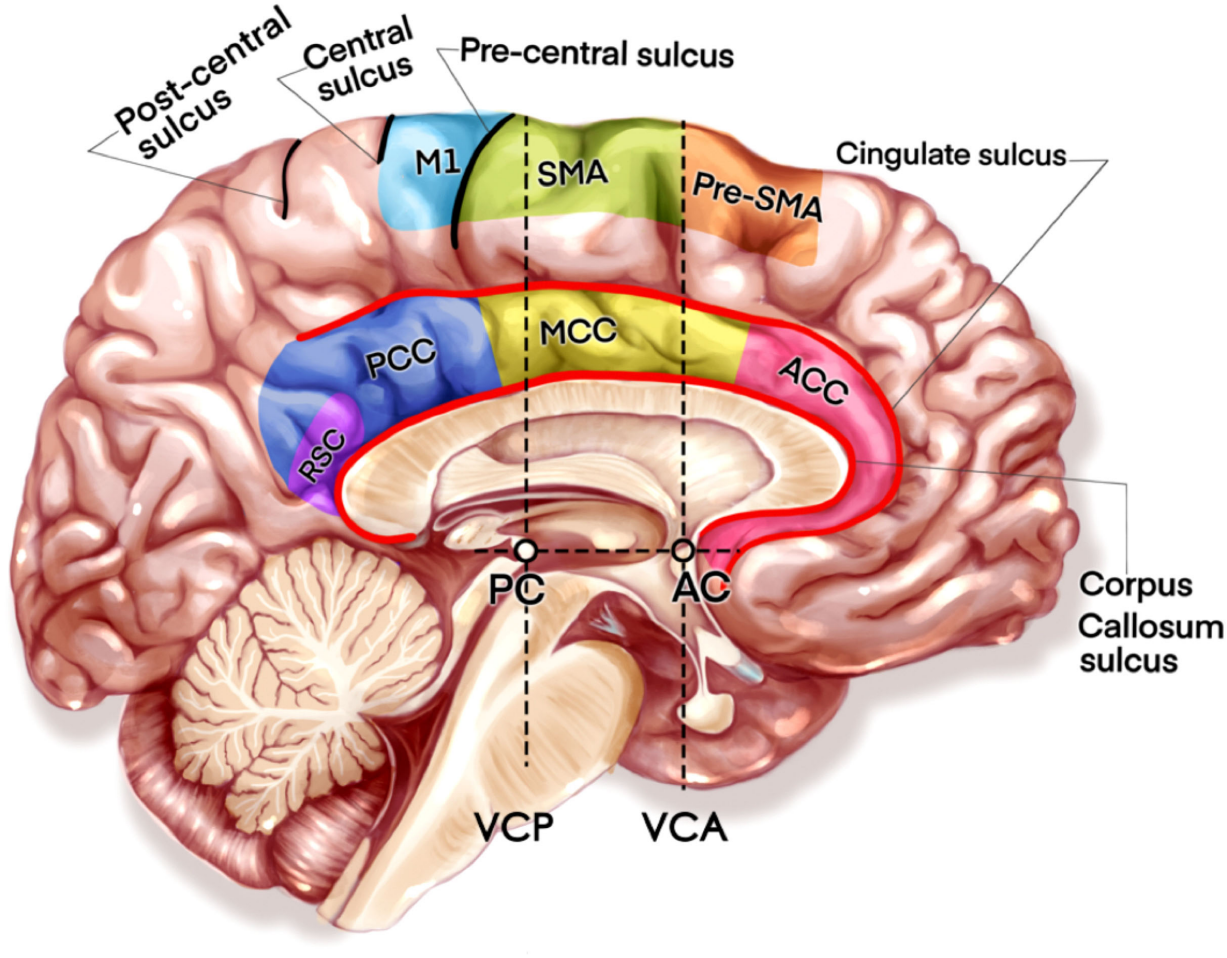
The illustration of four cingulate subregions and nearby motor related areas. AC, Anterior Commissure; PC, Posterior Commissure; VCA, Vertical Plane at the AC; VCP, VCA: Vertical Plane at the PC.

By reviewing the preoperative MRI images, a neurosurgeon made a preliminary diagnosis of cingulate gyrus glioma. Then a senior author judged the classification of the sub-region and growing types through whole perioperative MRI images and intraoperative videos. The division of four-subregion for CG gliomas corresponded to the Vogt model: ACC (Anterior Cingulate Cortex), MCC (Middle Cingulate Cortex), PCC (Posterior Cingulate Cortex), and RSC (Retrosplenial Cortex) (**Figure 1**). In addition, we defined 90% of the primary tumor restrained within the cingulate gyrus as pure CG glioma according to the growing presentation. If the growth of a partial tumor into the non-cingulate area was over 10%, but no more than 30%, we defined it as a diffuse CG glioma. If the lesion occupied two or more sub-regions of the CG, it would be classified as ACC+MCC, MCC+PCC, and ACC+MCC+PCC. Generally, the surgical plan was drawn up by a senior author after a preoperative discussion. The study and surgeries were approved by the Huashan IRB.

### Surgical approach and technique

We applied the transcortical approach or translongitudinal fissure approach (interhemispheric approach was also reported) for CG gliomas depending on sub-region and tumor growth type. Those lesions close to the functional cortex and subcortical fibers such as the pyramidal tract would preoperatively take BOLD (blood oxygen level–dependent) functional MRI and DTI (diffuse tensor imaging) examination DTI tractography was performed and a subcortical fiber close to the tumor was delineated on a data-processing workstation (Syngo MultiModality Workplace, Siemens®, Germany). Structural MRI and functional MRI (BOLD, DTI, and MRS) was integrated into the neuronavigation system (Medtronic®, United states). The intraoperative awake anesthesia or intravenous anesthesia were adopted on CG gliomas depending on the eloquent area or the primary nearby motor area after presurgical evaluation. When awake, craniotomy was manipulated, and the patient was in the supine position. A coronal or horseshoe-shaped incision was made based on boundary through navigation. After dura opening, theneurophysiological monitoring was working and Somatosensory Evoked Potentials (SSEP) was used to determine the central sulcus. The cortical electrode of the sensory evoked potential was placed at a position that did not hinder the surgical field, and a continuous monitoring of Motor Evoked Potential (MEP) by cortical electrical stimulation was performed. After checking the awake status of the patient, surgeons mapped the eloquent area by mapping tasks and cortical stimulation. In cases with intravenous anesthesia, and if necessary, transcortical monitoring of MEPs and subcortical electrical stimulation mapping were combined with DTI tractography for the motor-related areas.

### Extent of resection

The primary and postoperative tumor volumes were calculated on preoperative MRI and intraoperative MRI. If the intraoperative MRI was not available on the surgery date, MRI images within 24 hours after surgery were used to delineate tumor boundary. All images were processed in DICOM format with software Osirix® MD (version 10.0.1, http://www.osirix-viewer.com) by a radiologist and independently confirmed by a senior author. EOR was calculated with the formula: [(preoperative tumor volume - postoperative tumor volume)/preoperative tumor volume] × 100%. EOR would be compared among sub-region groups.

### Function evaluation and survival analysis

One to three days before the operation, all patients underwent comprehensive assessments of physical status, motor, and general cognitive function by using the Karnofsky Performance Scale (KPS), the Medical Research Council (MRC) Scale for Muscle Strength, and the Mini Mental State Examination (MMSE), respectively. In our study, subjects with muscle strength no better than grade III were defined as having motor dysfunction. Subjects with MMSE scores less than the cut-off value that depended on age and education were defined as having cognitive dysfunction (16). (Language function was assessed in detail by the Aphasia Battery for Chinese speakers (ABC) which is the Chinese standardized adaptation of the Western Aphasia Battery and included subscores for spontaneous speech, comprehension, repetition, and naming. The Aphasia Quotient (AQ) (range, 0–100) can be calculated from these items to reflect the global severity. Individuals with an AQ score of less than 93.8 were defined as having language dysfunction (17). Patients were evaluated at least at three-time points-preoperative period, subacute phase (i.e., 2 weeks and/or 1-month after surgery), and chronic phase (i.e., 3-months and/or 6-months after surgery). At each time point, clinical follow-ups and functional evaluations were both required. The evaluation was supervised by a senior author. The morbidity of short-term was defined as a new or worsening dysfunction in cognition, motor, or language evaluation compared with preoperative assessment. If the patient’s KPS was lower than a score of 60 in the subacute phase, the assessment was delayed until well cooperation was promised, but the latency time was no longer than 1 month. The long-term morbidity was defined as a permanent dysfunction that was already existing in the subacute phase and still presenting in the chronic phase. OS (overall survival) was defined as the days between surgery date and death.

### Statistical analysis

According to the four-subregion model, the patients would be divided into four groups. In fact, no cases of tumor within the RSC part were reported in the study, and three areas (ACC, MCC, and PCC) were analyzed as variables. Clinical characteristics including age, gender, tumor growth type, tumor laterality, histopathology, and molecular pathological markers were also converted into variables. All continuous variables were tested for normality. Homogeneity of variance was tested between different groups. Independent two-sample t-tests were used to compare continuous variables between two groups. One-way ANOVA was used to compare continuous variables among three groups. Dichotomous data were assessed using of the chi-squared test or Fisher’s exact test. In our study, short-term and long-term morbidities were regarded as dependent variables, and logistic regression analysis was used to observe OR (odds ratio) and 95% CI (confidence interval) (18). Overall survival was defined as the time between initial surgery and death. The Kaplan-Meier method was used to estimate the OS and Cox proportional hazards regression was used to assess the implications of various potential prognostic factors on OS. A probability level of 0.05 was accepted for the indication of statistical significance in double-sided testing. Statistical analyses were conducted with the use of commercial software (SPSS version 21).

## Result

### Patient demographics

The 95 adult cases of CG glioma had ages raging from 19 to 70 years old and consisted of 52 men and 43 women (**Table 1**). Fifty-four patients (56.84%) had left-sided gliomas. All patients were first hospitalized and preliminarily diagnosed with primary brain tumors in the outpatient clinic. Most of the patients (96.84%) had a preoperative Karnofsky Performance Scale score ≥ 90. Sixty-six patients (69.47%) were histopathologically diagnosed as WHO grade 2 gliomas and eleven patients (11.58%) were diagnosed as WHO grade 4 gliomas, but no H3K27M-mutant diffuse midline gliomas were reported. Most LGG cases were diffuse astrocytoma, IDH-mutation type, grade 2 and oligodendroglioma, 1p/19q co-deletion with IDH-mutation type, grade 2. The distribution of histopathology in different sub-regions showed no significant difference. The most common presenting chief complaint was seizures (56.84%), headache (33.68%), and lower limb weakness (21.05%). Among all LGG patients, fifty-three patients received adjuvant therapies. They received either radiotherapy (6.35%), chemotherapy (13.64%), or both (62.12%). All HGG patients received radiotherapy combined with chemotherapy according to the Stupp regimen.

**Table 1:** Summary of Clinical characteristics in 95 patients.

Parameter	Result (Total N=95)	
Age at diagnosis (years)		
Median	40	
Range	19-70	
Gender (cases)		
Male	52	
Female	43	
Cingulate gyrus glioma type (cases)		
Pure CG glioma (tumor restraining within cingulate gyrus)	65	
Diffuse CG glioma (tumor extending to non-cingulate gyrus)	30	
Side of tumor (cases)		
Left	54	
Right	41	
Main clinical symptoms (cases)		
Epilepsy	54	
Motor weakness	20	
Headache with nausea	32	
Disorder in memory	14	
WHO Grade (cases)		
2	66	
3	18	
4	11	
Pathology (cases)		
Astrocytoma, IDH-mutation, Grade 2		31	
Astrocytoma,IDH-mutation, Grade 3		6	
Oligodendroglioma,1p/19q Co-deletion, IDH-mutation, Grade 2		23	
Glioblastoma, IDH-wildtype, Grade 4		9	
Astrocytoma, IDH-wildtype, NEC, Grade 2		11	
Astrocytoma, IDH-wildtype, NEC, Grade 3		6	
Oligodendroglioma, 1p/19q Co-deletion, IDH-mutation, Grade 3		6	
Astrocytoma, IDH-mutation, Grade 4		2	
Pleomorphic xanthoastrocytoma		1	

### Distribution of the sub-regions

**Figure 2:**
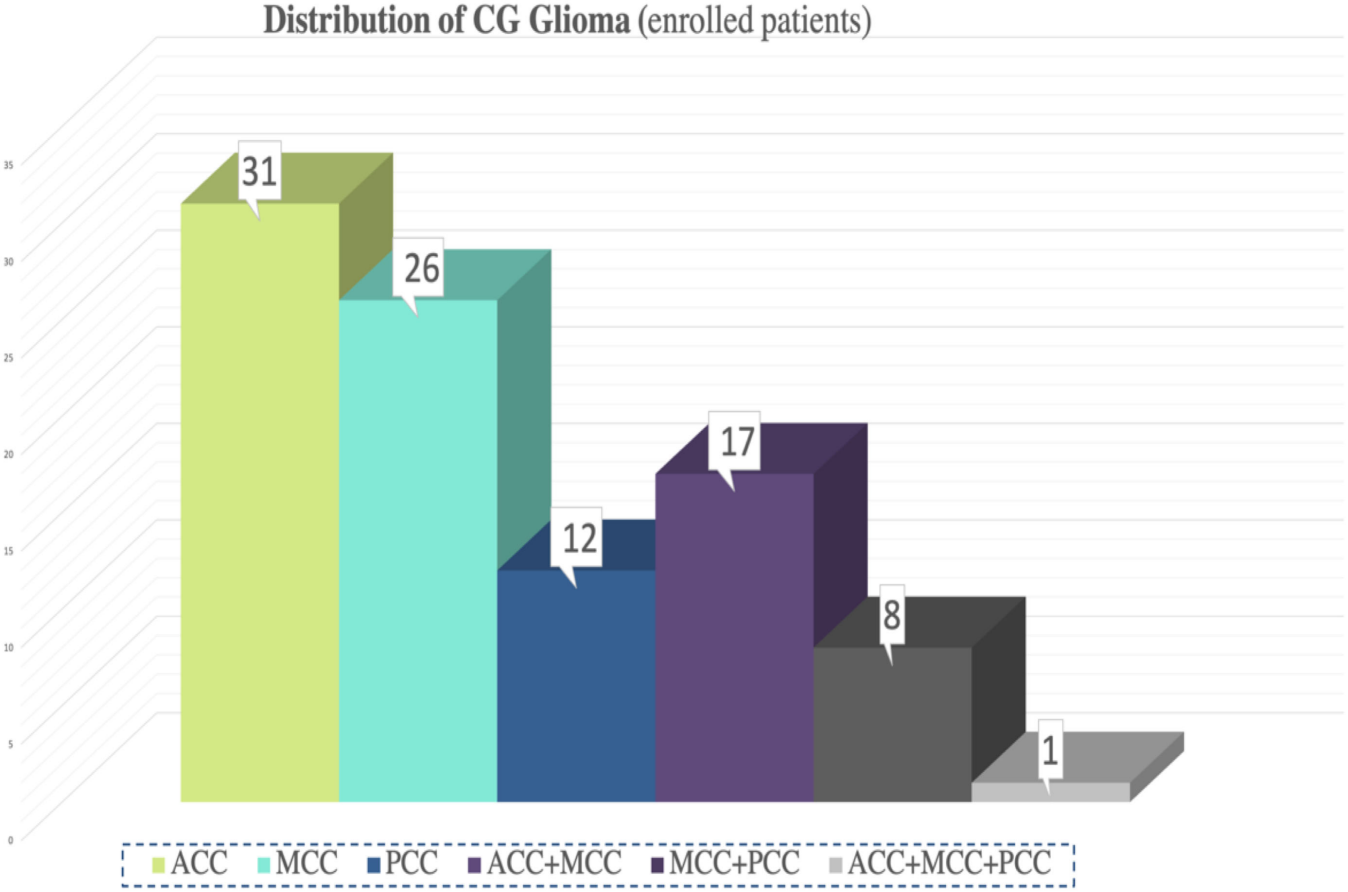
The distribution of cingulate gyrus glioma classified by sub-region.

According to extent of supracingular tumor, 65 patients (68.42%) had pure cingulate gliomas and 30 patients (31.58%) had diffuse cingulate gliomas. Among the diffuse cingulate gliomas, 19 invaded the contralateral hemisphere. There were 31 ACC cases, 26 MCC cases, 12 PCC cases, 17 ACC+MCC cases, 8 MCC+PCC cases, and one ACC+MCC+PCC case (**Figure 2**). Regarding the possibility of a single sub-region tumor invasion, MCC was the most prevalent (54.74%) followed by ACC. For those with pure cingulate gliomas, the biggest group was still MCC, totaling of 22 cases.

### Extent of resection

The results of EOR in CG gliomas are presented in **Table 2**. Among all patients, the range of tumor volume was 7.3 to 225.5 cm^3^ and the median was 40.26 cm^3^. In the pure CG glioma group, the average tumor volume was 38.25 cm3 while the average was 110.4 cm^3^ in the diffuse CG glioma group. A total of 83 patients (87.37%) received EOR ≥ 90% and 57 had pure CG gliomas. In the LGG group, 58 patients (87.88%) received EOR ≥ 90%. In the HGG group, 14 patients (48.28%) received EOR to 100%, which meant gross total resection (GTR). A significant difference was observed between cases of EOR ≥ 90% in the LGG group and cases of GTR in the HGG group (p<0.0001). The percentage of EOR ≥ 90% in the LGG group was higher than that of GTR cases in the HGG group. Regarding sub-regions, the cases of EOR ≥ 90% in the PCC group were significantly lower than those of the ACC (p=0.0004) and MCC cases (p=0.0045). The average EOR of ACC, MCC, and PCC groups were 97.38%, 94.77 and 94.32%.

**Table 2:** Summary of tumor volume and EOR (extent of resection) in 95 patients.

Index		All patients	Group					LGG	HGG
			ACC*	MCC*	PCC*	Pure CG	Diffuse CG		
Median tumor volume (cm^3^)		40.26	53.29	49.75	37.71	37.19	87.06	41.10	38.73
	≥90% (Cases)	83	45	45	17	57	26	58	14 (=100%)
	<90% (Cases)	12	4	7	12	8	4	8	15 (<100%)
**EOR Values**	Mean (%)	96.06	97.38	94.77	94.32	96.04	96.13	95.62	95.73
	Median (%)	100.00	100.00	94.71	93.42	100.00	100.00	100	99.76
	Range (%)	83.96-100.00	83.96-100.00	84.01-100.00	85.40-100.00	83.96-100.00	84.01-100.00	81.27-100.00	82.65-100.00

*Subregion in table included all patients where tumor involved in (either alone or in combination with other zones).

All patients, n = 95; Tumor involved in ACC, n = 49; Tumor involved in MCC, n = 52; Tumor involved in PCC, n = 29.

### Function outcome

**Table 3** presents the frequency of deficits among all patients classified in groups. MCC tumor patients were significantly associated with short-term morbidity in either language function (OR:8.44, 95%CI 2.30-26.16, p=0.0007) or motor function (OR:5.83, 95%CI 1.53-21.35, p=0.0129) compared with ACC cases. If we regarded language, motor and cognition disorder as postoperative deficits, we found that 25 of 31 pure ACC patients (80.65%) can escape from any short-term deficit and nearly 90% from permanent morbidity. In total, 16 pure MCC patients (61.54%) and 6 pure PCC patients (50%) were reported short-term dysfunctions under standard surgical approaches and techniques. However, the proportion of long-term dysfunction decreased to 15.38% and 33.33% for MCC and PCC respectively. No significant difference was observed in long-term morbidity among the sub-regions. To compare transboundary tumors or multiple-subarea tumors, **Table 4** presents the relationship between postoperative morbidity and the sub-regions* affected by tumors. The frequency of distribution in **Table 4** included all cases whose tumors involved the subarea, either alone or combined with other sub-area. For example, the MCC* group included not only pure MCC tumors but also infiltrated transboundary tumors into ACC or PCC. Through statistical analysis, 23 patients (44.23%) and 17 patients (32.69%) in the MCC* group reported language and motor deficits. However, only four (4.21%) and six patients (6.32%) in this group still presented either language or motor dysfunctions in long-term follow-ups. The percentage of recovery was 82.6% and 41.18% for language and motor dysfunctions. Although previously seizure attacks were reported as the most common chief complaint, only 10 patients (10.52%) in short-term and four patients (4.21%) in follow-ups long-term claimed seizure attack.

**Table 3:** Summary of function deficits and clinical symptoms in classified groups.

Distribution	No. of patients	Postoperative short-term (within one month) morbidity cases				
		Language	Motor	Language with motor	Cognition	Seizure
ACC	31	4	3	2	2	1
MCC	26	15	10	9	4	3
PCC	12	3	4	1	3	0
ACC+MCC	17	5	5	1	1	3
MCC+PCC	8	3	2	2	3	0
ACC+MCC+PCC	1	0	0	0	0	0
		**Postoperative long-term (within six month) morbidity cases**				
ACC	31	3	1	0	0	0
MCC	26	2	3	1	1	0
PCC	12	2	2	0	0	0
ACC+MCC	17	0	2	0	1	2
MCC+PCC	8	2	1	0	1	0
ACC+MCC+PCC	1	0	0	0	0	0

**Table 4:** Summary of relationship between function deficits and distribution of sub-region.

Parameter (Subregion involved)	Postoperative short-term (within one month) morbidity				
	Language	Motor	Language with motor	Cognition	Seizure
Pure CG glioma					
ACC* n=25	5	4	2	2	3
MCC* n=39	17	13	10	7	4
PCC* n=19	5	5	3	4	0
All CG glioma					
ACC* n=49	9	8	2	3	4
MCC* n=52	23	17	12	8	6
PCC* n=29	6	6	3	6	0
**Parameter (Subregion involved)**		**Postoperative long-term (within six months) morbidity**				
	**Language**	**Motor**	**Language with motor**	**Cognition**	**Seizure**
Pure CG glioma					
ACC* n=25	2	2	0	1	2
MCC* n=39	4	6	1	3	2
PCC* n=19	3	2	0	2	0
All CG glioma					
ACC* n=49	3	3	0	1	2
MCC* n=52	4	6	1	3	2
PCC* n=29	4	3	0	2	0

Subregion* in table included all patients whose tumor involved the area (either alone or in combination with other areas).

**Table 5** presents the function outcomes in the dominant hemisphere. Whatever the type of glioma, pure CG or diffuse type, tumors in the dominant hemisphere were significantly associated with language dysfunction or cognitive dysfunction, either at short-term (OR:6.70, 95% CI: 2.21-19.1, p=0.0006) or long-term morbidity (OR:6.35, 95% CI: 1.77-99.9, p=0.0111). Those patients who suffered from language dysfunction combined with motor dysfunction in the short term were almost all dominant hemisphere gliomas. However, this type of combined dysfunction can almost relieve until recovery (92.86%).

**Table 5:** Summary of relationship between function deficits and dominant hemisphere.

Parameter (Tumor side)	Postoperative short-term (within one month) morbidity						
	Language	Motor		Language with motor		Cognition	Seizure
Pure CG glioma							
Dominant hemisphere	20	13		11		9	2
Non-dominant hemisphere	2	4		1		0	3
All CG glioma							
Dominant hemisphere n=58	26	18		13		13	4
Non-dominant hemisphere n=37	4	6		1		0	3
**Parameter (Tumor side)**		**Postoperative long-term (within six months) morbidity**						
	**Language**	**Motor**		**Language with motor**		**Cognition**	**Seizure**
Pure CG glioma							
Dominant hemisphere	7	4		1		3	1
Non-dominant hemisphere	0	3		0		0	1
All CG glioma							
Dominant hemisphere n=58	9	5		1		3	1
Non-dominant hemisphere n=37	0	4		0		0	1
**Language morbidity for all CG glioma**	**OR**		**95% CI**		**P value**		
Short-term (Dominant hemisphere)	6.703		2.21-19.1		0.0006		
Long-term (Dominant hemisphere)	6.353		1.77-99.9		0.0111		

**Table 6** presents the results of multivariate logistic regression model for postsurgical deficit. The factors contributing to a short-term dysfunction were age, sub-region of MCC and dominant hemisphere. If the tumor was in the midcingulate cortex (OR:3.01, 95% CI: 1.27-7.13, p<0.05) or dominant hemisphere (OR:3.44, 95% CI: 1.27-9.30, p<0.05), function deficits were more likely to happen. The only factor associated with a long-term dysfunction was age. The older cases were, the worse prognosis of function turned out, regardless of the short-term or long-term type of observation.

**Table 6:** Analysis of multivariate logistic regression for postsurgical deficit.

Factors	Short-term			Long-term		
	OR	95%CI	P value	OR	95%CI	P value
Tumor growing type (Diffuse tumor)	1.745	0.641-4.746	0.276	0.698	0.17-2.867	0.618
Sub-region (MCC)	3.014	1.274-7.129	0.020	1.454	0.423-4.994	0.552
Dominant hemisphere	3.442	1.274-9.296	0.015	2.433	0.704-8.406	0.16
Grade (LGG)	0.872	0.312-2.437	0.794	0.527	0.423-4.994	0.374
Age	1.046	1.007-1.087	0.021	1.062	1.010-1.116	0.02
Extent of Resection (LGG>90% or HGG=100%)	0.004	0.000-41.613	0.242	2.887	0.706-11.802	0.14

### Survival analysis

In this study, overall survival data were available among all patients. At the last follow-up, 19 patients (20.00%) deceased, and 13 of (68.42%) were HGG. A significance was observed in longer survival in the LGG group (median not reached, mean [at last follow-up] 75.3 months, 95% CI 71.1–79.5 months) compared with HGG group in **Table 7** (median 59.5 months, mean 45.8 months, 95% CI 36.4–55.2 months, p=0.004). Among the HGG group, grade 3 glioma presented a better prognosis than that of grade 4 glioma. IDH-mutant and EOR significantly correlated with OS. IDH-mutant group (median not reached, mean [at last follow-up] 76.6 months, 95% CI 73.2–80.1 months) reported a significantly better prognosis than that of the IDH-wildtype group (median 35.3 months, mean 37.2 months, 95% CI 27.6–46.8 months, p<0.0001). IDH-mutation showed no significant difference among the classified sub-regions. We defined EOR ≥ 90% in the LGG group and GTR in the HGG group as the goal of EOR. Patients who achieved the goal of EOR (median not reached, mean [at last follow-up] 71.9 months, 95% CI 66.8–77.0 months) had a significantly better OS than those who did not achieve the goal (median 59.5 months, mean [at last follow-up] 50.0 months, 95% CI 40.0–60.1 months, p=0.006). The type of diffuse glioma could lead to a worse prognosis (HR: 3.91, 95% CI: 1.09-14.04, p=0.037). The sub-regions that we were concerned about, were not influencing factors of OS.

**Table 7:** Multivariate analyses of overall survival outcome in CG patients.

Multivariate analysis	P Value	HR	95%CI	
			Lower	Upper
Age	0.180	0.975	0.939	1.012
Tumor growing type (Diffuse type)	0.037	3.911	1.089	14.038
Sub-region (Non-MCC)	0.244	0.498	0.154	1.608
WHO Grade (LGG)	0.004	0.146	0.04	0.541
IDH (Mutant)	<0.0001	0.031	0.005	0.185
Tumor volume	0.944	0.999	0.978	1.021
Extent of resection (LGG>90% or HGG=100%)	0.006	0.212	0.066	0.678

## Discussion

### Morbidity and cingulate anatomy

In the results’ part, MCC cases were significantly associated with short-term morbidity in either language or motor function. In our opinion the contributors to postoperative morbidity in this study are damages in cingulate cortex and subcortical structures. Therefore, it is necessary to review the cingulate anatomy. **Figure 1** presents a critical medial hemispheric motor related area in midsagittal brain view. Previous studies provided evidence about the role of MCC regulation in mediating skeletomotor functions (12, 19). The subcortical connection from MCC to M1 or SMA may play a role in movement planning and even speech initiation (20). Although some neurosurgeons hold the point FAT (frontal aslant tract), related deficits are usually temporary and invariably recover (21). Others still support the practice of awake anesthesia combined with brain mapping in SMA-cingulum-corpus glioma resection (22). In our study, the MCC tumor with ACC boundary was usually adopted using intravenous anesthesia with continuous MEP monitoring while tumors with more posterior boundary were performed under awake anesthesia due to the necessity to protect M1. ACC tumors presented a remarkable function outcome and almost all were free from permanent morbidity. ACC were regarded as activators of pain processing with projections to MITN (midline and intra laminar thalamic nuclei) (23). Recently, clinicians realized that ACC is a key region for acute pain perception as well as the development of neuropathic pain that contributes to the unpleasantness of pain, salience, and regulation of emotional information (24). The limitation of our retrospective study is in its difficulty to judge transient pain experience as dysfunction in long-term follow-up, but neuropathic pain provides a new direction in the observation of dysfunction. Recently, PCC was regarded as the hub of the default mode network (DMN), participating in the initial deactivated function and in engaging in spontaneous cognition (25). A recent imaging study on brain glioma showed that neurological profiles, including verbal fluency, verbal memory, and visual memory, are longitudinally associated with spatial features of the connectome, which is mainly within the DMN (26). Another study found that the surgical tissue changes of brain glioma, such as resection cavities or edema, have strong negative impacts on DMN nodes that result in bad performance in language processing and verbal working memory (27). Our result demonstrates that postsurgical PCC cases can suffer from deficits in language or cognition, but it is still ambiguous whether PCC glioma can lead to disorders with alterations of spatial connectomic metrics in DMN. The cingulum is not a unitary fasciculus, but a united bundle comprised with numbers of association fibers connecting ACC to PCC, that associate with frontal and parietal cortex and extend to the anterior thalamus region and parahippocampal region (28). Although some reports imply that extending the cingulum lesion with a second surgery can have a more favorable outcome, a study of major depression still found that smaller lesion volumes are associated with better results (29). Despite an injury to combined association fibers, function disturbance might not be clearly observed by clinical evaluation. In our study, essential hubs or segments of cingulum might support a crucial neurological or cognition function and injuries to hubs that cause profound cut-off performance in evaluation.

### Surgical approach and management

The surgical strategies and management are summarized in the **Table 8** based on the experience gained from our surgeries. The enumerated plan aimed at classifying pure CG gliomas into sub-regions. The surgical preparation comprises body position, skin incision, surgical approach, functional evaluation, and fMRI modalities. The recommended surgical equipment includes the method of anesthesia, the pattern of neurophysiological monitoring, mapping strategies, and the stimulator. In our study, the percentage (56.84%) of preoperative seizure attacks among all cingulate cases was higher than that of the cohort of 587 glioma cases reported (22.3%) by Yu (30). The study identified preoperative epilepsy as an independent risk factor of postoperative epilepsy in patients with HGG. Based on this, the management of anti-epilepsy in cingulate cases is necessary, including the use of regular and anti-epilepsy drugs (AEDs). The recommendation of an intraoperative mapping strategy is conservative and prevents patients from intraoperative epilepsy.

**Table 8:** Recommendable regimen of surgical approach and techniques for pure CG glioma in different sub-region.

	ACC	ACC and MCC boundary	MCC	PCC
Common chief complaint	Seizure attack	Seizure attack	Motor weakness	Motor weakness
**Surgical Planning**				
**Position**	Supine position with head flexed	Supine position with head flexed	Supine position with upper body raised	Prone position with neck flexed
**Incision**	Coronal incision	Horseshoe shaped incision	Horseshoe shaped incision	U-shaped incision or paramedian straight incision
**Surgical approach**	Trans-longitudinal fissure approach	Trans-longitudinal fissure approach	Trans medial frontal cortical approach	Trans precuneus cortical approach
**Preoperative Preparation**				
**Neurological evaluation**	Necessary	Necessary	Necessary	Necessary
**DTI tractography**	Depend on evaluation	Necessary (Motor)	Necessary (Language + Motor)	Necessary (Motor)
**BOLD**	Depend on evaluation	Necessary (Motor)	Necessary (Language + Motor)	Necessary (Motor)
**Intraoperative equipment**				
**Anesthesia**	Intravenous anesthesia	Intravenous anesthesia	Awake anesthesia	Intravenous anesthesia or Awake anesthesia
**Electrophysiology**	Not applicable	Necessary (MEP monitoring)	Necessary (Transcortical MEP +SSEP)	Necessary (MEP monitoring + SSEP, or transcortical MEP + SSEP for awake anesthesia
**Stimulator**	Not applicable	Not applicable	Ojemann stimulator (5 mm-interval bipolar electrodes, current-constant bipolar square wave, 1-ms wave width, 60 Hz frequency)	
**Mapping strategy**	Not applicable	Transcortical MEP monitoring, strip electrode being put on the posterior side of surgical field	Stimulate the paracentral lobule until reliable motor responses were induced. Language mapping current was set to the same intensity	Stimulate the paracentral lobule until reliable motor/sensory responses were induced
**Concerned blood vessels**	ACA, Orbital-frontal A., Pericallosal A., Callosomarginal A. (Anteromedial frontal branch)	Pericallosal A., Callosomarginal A. (Intermedial frontal branch)	Pericallosal A., Callosomarginal A. (Intermedial and Posteromedial frontal branches)	Pericallosal A., PCA (Parieto-occipital branch and Paracentral branch)

MEP, motor evoked potential; SSEP, Somatosensory evoked potential; ACA, anterior cerebral artery; PCA, posterior cerebral artery.

### Recovery and survival influencing factors

The factors contributing to short-term dysfunction are age, sub-region, and the dominant side. The above anatomy of the cingulate gyrus explains how the location of the sub-region affects the performance of short-term function. Regarding the factors of the dominant side, a study of direct electrical stimulation (DES) showed that the stimulation of SMA, Pre-SMA, and the cingulate motor area (part of MCC as described) on the dominant side can cause speech-related responses like speech arrest or complex responses such as a disturbance in motor selection (31).

In our study, our results infer that the dominant side of cingulate tumors is closely related to postsurgical function deficit. However, some recent studies have shown that subcircuits of the sociocognitive subsystem are conducted by non-dominant cingulum and PCC through multimodal imaging analysis (32). One limitation of our study is the evaluation tools for cognition outcomes that were uncapable of complex assessments on sociocognitive or mentalization function. The only factor associated with both transient and permanent dysfunctions was age. Based on our results, the older cases had the worse recovery performance. The potential of neuroplasticity has been proved to play a vital role in function compensation in many studies. One explanation for recovery might be the reorganization of function networks that are coordinated by remote networks. A report of DES study on eloquent area glioma found that areas are immediately recruited around the lesion after resection and remapping can involve distant regions in the ipsilateral hemisphere (33). However, this mechanism of reorganization is influenced by ageing. Using rTMS (repetitive transcranial magnetic stimulation) protocols, studies on neuroplasticity reported ageing might be associated with a reduced capacity for motor cortical plasticity (34). It is also claimed that potential for plasticity differ in cortex and subcortical fibers. Indeed, subcortical neuroplasticity is more unsubstantial and lower than cortical level, which is the same repairing effect as that of rehabilitation therapy. Therefore, to avoid severe function deficits in cingulate cases, applying surgical techniques combined with indispensable equipment is still the wisest strategy.

Our result has shown that influencing factors which affect overall survival are tumor grade, molecular marker IDH, and extent of resection. IDH marker and EOR are both independent predictors of survival in CG glioma. Based on the study of IDH, a consensus has been reached on the correlation between IDH-mutation type and better survival outcomes (35). The significance of our study is to reclaim the homogeneity of IDH presentation and the necessity to test the molecular marker IDH despite the infrequency of CG glioma. the histopathological distribution in various sub-regions showed no significant difference. If divided into IDH-mutation astrocytoma and IDH-mutation oligodendroglioma, the samples of sub-group would be too small to be persuasive on comparative results. Even so, whether the IDH is mutant or not is sufficiently profound to adjust surgical extent of cingulate cortex for neurosurgeons. A new rapid detection of IDH enables surgeons to determine real-time IDH mutation intraoperatively (36).

In our study, EOR is one of the most significant determinants of overall survival. EOR over 90% in the LGG group and GTR in the HGG group are likely to promise a better prognosis than those with no EORs achievement. Based on the latest clinical practice guidelines for the management of adult diffuse gliomas in China (37), it is commonly inadvisable to achieve total resection at the expense of function impairment for grade 2 gliomas. However, for WHO grade 3-4 gliomas, it is suggested to perform maximal resection of the contrast-enhanced tumor with the additional maximum resection of the non-contrast-enhanced tumor when safely feasible. Professor Berger suggested low-grade gliomas had a better 10-year survival when extent of resection was greater than 90% and patients with high-grade gliomas experienced improved survival and quality of life with maximal resection (38). Therefore, we set 90% of EOR in LGGs and GTR in HGGs as criterion among CG glioma patients. Since the presentation of a molecular marker is objective, the most effective effort to promise better survival is still to achieve EOR as recommended for both LGG and HGG. Recent studies have demonstrated that a safe and maximal tumor resection improve symptomatic management, quality of life, and OS in glioma patients. Like the functional outcome of ACC cases, we present EOR can be completely expanded to a safe margin with the aid of surgical equipment. As for MCC cases, as long as a full monitoring of the eloquent area is adopted, a maximal safe tumor resection is still the target of the operation. It is difficult to define a certain number for EOR on CG glioma, but the above surgical techniques and management according to sub-regions can be advised to define a safe and maximal resection.

## Conclusion

The postoperative neurological deficits of the cingulate gyrus are associated with the sub-regions of the tumor, dominant hemisphere, and age. MCC gliomas are most likely to suffer from postoperative language or motor dysfunction in the short term. Cingulate gyrus glioma on the dominant side leads to language and cognition dysfunction more likely than that on the non-dominant side. The clinical value is considered to be pertinent surgical managements and equipment for each subregion. Maximizing advisable EOR during operation is still a promising way to prolong survival for cingulate gyrus tumors in all sub-regions.

## Data availability statement

The raw data supporting the conclusions of this article will be made available by the authors, without undue reservation.

## Ethics statement

This study was reviewed and approved by Huashan Hospital Institutional Review Board. The patients/participants provided their written informed consent to participate in this study. Written informed consent was obtained from the individual(s) for the publication of any potentially identifiable images or data included in this article.

## Author contributions

JW Designed the study, Performed surgeries, Analyzed the data, Revised the manuscript. FG Designed the study, Gathered and Analyzed the data, Drafted the manuscript. LJ Edited and revised the manuscript. QS Assisted in surgeries and patient caring. ZY Revised medical image data. HC Revised pathological data. All authors contributed to the article and approved the submitted version.

## Funding

This study was supported by Shanghai Shenkang Hospital Development Center (grant no. SHDC12018114) and Science and Technology Commission of Shanghai Municipality (grant no. 22S31905400).

## Conflict of interest

The authors declare that the research was conducted in the absence of any commercial or financial relationships that could be construed as a potential conflict of interest.

## Publisher’s note

All claims expressed in this article are solely those of the authors and do not necessarily represent those of their affiliated organizations, or those of the publisher, the editors and the reviewers. Any product that may be evaluated in this article, or claim that may be made by its manufacturer, is not guaranteed or endorsed by the publisher.

## Abbreviations

CG, cingulate gyrus; EOR, extent of resection; GTR, gross total resection; OS, overall survival; MRI, magnetic resonance imaging; DTI, Diffusion tensor imaging; MRS, magnetic resonance spectroscopy; BOLD, blood oxygen level-dependent; OR, odds ratio; CI, confidence interval; HR, hazard rate; ACC, anterior cingulate cortex; MCC, midcingulate cortex; PCC, posterior cingulate cortex; RSC, retrosplenial cortex.

## References

[1] Reep R. Relationship between prefrontal and limbic cortex: A comparative anatomical review. Brain Behav Evol (1984) 25(1):5–80. doi: 10.1159/000118849 6398115

[2] Vogt BA Pandya DN Rosene DL. Cingulate cortex of the rhesus monkey: I. Cytoarchitect Thalamic Afferents. J Comp Neurol (1987) 262(2):256–70. doi: 10.1002/cne.902620207 3624554

[3] Weininger J Roman E Tierney P Barry D Gallagher H Murphy P. Papez’s forgotten tract: 80 years of unreconciled findings concerning the thalamocingulate tract. Front Neuroanat (2019) 13:14. doi: 10.3389/fnana.2019.00014 30833890 PMC6388660

[4] Rolls ET O’Doherty J Kringelbach ML Francis S Bowtell R McGlone F. Representations of pleasant and painful touch in the human orbitofrontal and cingulate cortices. Cereb Cortex (2003) 13(3):308–17. doi: 10.1093/cercor/13.3.308 12571120

[5] Morecraft RJ Van Hoesen GW. Frontal granular cortex input to the cingulate (M3), supplementary (M2) and primary (M1) motor cortices in the rhesus monkey. J Comp Neurol (1993) 337(4):669–89. doi: 10.1002/cne.903370411 8288777

[6] Luerding R Weigand T Bogdahn U Schmidt-Wilcke T. Working memory performance is correlated with local brain morphology in the medial frontal and anterior cingulate cortex in fibromyalgia patients: Structural correlates of paincognition interaction. Brain (2008) 131:3222–31. doi: 10.1093/brain/awn229 18819988

[7] Vogt BA. Pain and emotion interactions in subregions of the cingulate gyrus. Nat Rev Neurosci (2005) 6(7):533–44. doi: 10.1038/nrn1704 PMC2659949 15995724

[8] Tate MC Kim CY Chang EF Polley MY Berger MS. Assessment of morbidity following resection of cingulate gyrus gliomas. Clin Article. J Neurosurg (2011) 114(3):640–7. doi: 10.3171/2010.9.JNS10709 20932098

[9] von Lehe M Schramm J. Gliomas of the cingulate gyrus: Surgical management and functional outcome. Neurosurg Focus (2009) 27(2):E9. doi: 10.3171/2009.6.FOCUS09104 19645564

[10] Heilbronner SR Haber SN. Frontal cortical and subcortical projections provide a basis for segmenting the cingulum bundle: Implications for neuroimaging and psychiatric disorders. J Neurosci (2014) 34(30):10041–54. doi: 10.1523/JNEUROSCI.5459-13.2014 PMC4107396 25057206

[11] Beckmann M Johansen-Berg H Rushworth MF. Connectivity-based parcellation of human cingulate cortex and its relation to functional specialization. J Neurosci (2009) 29(4):1175–90. doi: 10.1523/JNEUROSCI.3328-08.2009 PMC6665147 19176826

[12] Shima K Aya K Mushiake H Inase M Aizawa H Tanji J. Two movement-related foci in the primate cingulate cortex observed in signal-triggered and self-paced forelimb movements. J Neurophysiol (1991) 65(2):188–202. doi: 10.1152/jn.1991.65.2.188 2016637

[13] Morecraft RJ Stilwell-Morecraft KS Rossing WR. The motor cortex and facial expression: New insights from neuroscience. Neurologist (2004) 10(5):235–49. doi: 10.1097/01.nrl.0000138734.45742.8d 15335441

[14] Oszvald A Quick J Franz K Guresir E Szelenyi A Vatter H. Resection of gliomas in the cingulate gyrus: Functional outcome and survival. J Neurooncol (2012) 109(2):341–8. doi: 10.1007/s11060-012-0898-0 22660921

[15] Nakajima R Kinoshita M Nakada M. Simultaneous damage of the cingulate cortex zone ii and fronto-striatal circuit causes prolonged selective attentional deficits. Front Hum Neurosci (2021) 15:762578. doi: 10.3389/fnhum.2021.762578 35002655 PMC8740164

[16] Li H Jia J Yang Z. Mini-mental state examination in elderly Chinese: A population-based normative study. J Alzheimers Dis (2016) 53(2):487–96. doi: 10.3233/JAD-160119 27163822

[17] Lu J Wu J Yao C Zhuang D Qiu T Hu X. Awake language mapping and 3-Tesla intraoperative mri-guided volumetric resection for gliomas in language areas. J Clin Neurosci (2013) 20(9):1280–7. doi: 10.1016/j.jocn.2012.10.042 23850046

[18] Hameed NUF Qiu T Zhuang D Lu J Yu Z Wu S. Transcortical insular glioma resection: Clinical outcome and predictors. J Neurosurg (2018) 131(3):706–16. doi: 10.3171/2018.4.JNS18424 30485243

[19] Morecraft RJ Van Hoesen GW. Cingulate input to the primary and supplementary motor cortices in the rhesus monkey: Evidence for somatotopy in areas 24c and 23c. J Comp Neurol (1992) 322(4):471–89. doi: 10.1002/cne.903220403 1383283

[20] Dick AS Garic D Graziano P Tremblay P. The frontal aslant tract (Fat) and its role in speech, language and executive function. Cortex (2019) 111:148–63. doi: 10.1016/j.cortex.2018.10.015 PMC6461388 30481666

[21] Young JS Lee AT. Chang EF. A Rev Cortical Subcortical Stimulation Mapp Language. Neurosurg (2021) 89(3):331–42. doi: 10.1093/neuros/nyaa436 PMC8492609 33444451

[22] Klitsinikos D Ekert JO Carels A Samandouras G. Mapping and anatomo-surgical techniques for sma-Cingulum-Corpus callosum gliomas; how I do it. Acta Neurochir (Wien) (2021) 163(5):1239–46. doi: 10.1007/s00701-021-04774-7 PMC8053665 33779836

[23] Vogt BA Rosene DL Pandya DN. Thalamic and cortical afferents differentiate anterior from posterior cingulate cortex in the monkey. Science (1979) 204(4389):205–7. doi: 10.1126/science.107587 107587

[24] Moon HC Park YS. Optogenetic stimulation of the anterior cingulate cortex modulates the pain processing in neuropathic pain: A review. J Mol Neurosci (2022) 72(1):1–8. doi: 10.1007/s12031-021-01898-4 34505976

[25] Buckner RL DiNicola LM. The brain’s default network: Updated anatomy, physiology and evolving insights. Nat Rev Neurosci (2019) 20(10):593–608. doi: 10.1038/s41583-019-0212-7 31492945

[26] Saviola F Zigiotto L Novello L Zaca D Annicchiarico L Corsini F. The role of the default mode network in longitudinal functional brain reorganization of brain gliomas. Brain Struct Funct (2022). doi: 10.1007/s00429-022-02490-1 PMC9653323 35460446

[27] Kocher M Jockwitz C Caspers S Schreiber J Farrher E Stoffels G. Role of the default mode resting-state network for cognitive functioning in malignant glioma patients following multimodal treatment. NeuroImage Clin (2020) 27:102287. doi: 10.1016/j.nicl.2020.102287 32540630 PMC7298724

[28] Bubb EJ Metzler-Baddeley C Aggleton JP. The cingulum bundle: Anatomy, function, and dysfunction. Neurosci Biobehav Rev (2018) 92:104–27. doi: 10.1016/j.neubiorev.2018.05.008 PMC6090091 29753752

[29] Steele JD Christmas D Eljamel MS Matthews K. Anterior cingulotomy for major depression: Clinical outcome and relationship to lesion characteristics. Biol Psychiatry (2008) 63(7):670–7. doi: 10.1016/j.biopsych.2007.07.019 17916331

[30] Yu Z Zhang N Hameed NUF Qiu T Zhuang D Lu J. The analysis of risk factors and survival outcome for Chinese patients with epilepsy with high-grade glioma. World Neurosurg (2019) 125:e947–e57. doi: 10.1016/j.wneu.2019.01.213 30763739

[31] Chassagnon S Minotti L Kremer S Hoffmann D Kahane P. Somatosensory, motor, and Reaching/Grasping responses to direct electrical stimulation of the human cingulate motor areas. J Neurosurg (2008) 109(4):593–604. doi: 10.3171/JNS/2008/109/10/0593 18826345

[32] Duffau H. Stimulation mapping of white matter tracts to study brain functional connectivity. Nat Rev Neurol (2015) 11(5):255–65. doi: 10.1038/nrneurol.2015.51 25848923

[33] Sarubbo S Le Bars E Moritz-Gasser S Duffau H. Complete recovery after surgical resection of left wernicke’s area in awake patient: A brain stimulation and functional mri study. Neurosurg Rev (2012) 35(2):287–92. doi: 10.1007/s10143-011-0351-4 21947553

[34] Ridding MC Ziemann U. Determinants of the induction of cortical plasticity by non-invasive brain stimulation in healthy subjects. J Physiol (2010) 588(Pt 13):2291–304. doi: 10.1113/jphysiol.2010.190314 PMC2915507 20478978

[35] Hartmann C Meyer J Balss J Capper D Mueller W Christians A. Type and frequency of Idh1 and Idh2 mutations are related to astrocytic and oligodendroglial differentiation and age: A study of 1,010 diffuse gliomas. Acta Neuropathol (2009) 118(4):469–74. doi: 10.1007/s00401-009-0561-9 19554337

[36] Diplas BH Liu H Yang R Hansen LJ Zachem AL Zhao F. Sensitive and rapid detection of tert promoter and idh mutations in diffuse gliomas. Neuro Oncol (2019) 21(4):440–50. doi: 10.1093/neuonc/noy167 PMC6422442 30346624

[37] Jiang T Nam DH Ram Z Poon WS Wang J Boldbaatar D. Clinical practice guidelines for the management of adult diffuse gliomas. Cancer Lett (2021) 499:60–72. doi: 10.1016/j.canlet.2020.10.050 33166616

[38] Hervey-Jumper SL Berger MS. Maximizing safe resection of low- and high-grade glioma. J Neurooncol (2016) 130(2):269–82. doi: 10.1007/s11060-016-2110-4 27174197

