# Supplementary material for: Surgical techniques and function outcome for cingulate gyrus glioma, how we do it
Source: Front Oncol. 2022 Sep 26;12:986387. doi: 10.3389/fonc.2022.986387 (PMC9549335; doi:10.3389/fonc.2022.986387)
Supplement: Supplementary file 4 [file DataSheet_1.docx]

***Illustrative Cases***

How we perform the surgeries

In order to recommend the surgical approach and techniques, we applied as standard, three illustrative cases of pure CG gliomas were demonstrated in detail.

Illustrative case 1

The patient was a 40-year-old male. A routine examination of head MRI showed lesion in his left anterior cingulate cortex. He had experienced intermittent mild headache for 2 months. (Figure 3.) The tumor was maily in subgenual part of the ACC. To achieve maximal protection for cingulum and frontal aslant tract, translongitudinal fissure approach was chosen. Cortical incision was located on the medial side of the superior frontal gyrus. The cingulate sulcus can be figured by pericallosal artery. Neuronavigation was used to identify and resect deeper ends of the tumor until lamina terminalis reached. Histopathology confirmed diagnosis of “anaplastic oligodendroglioma, IDH-mutant and 1p/19q-codeleted”. Adjuvant chemo-therapy was adopted according to Stupp’s regimen for six months. During whole perioperative period, no morbidities happened. The patient quick transition back to normal life

Illustrative case 2

The patient was a 41-year-old male. The patient underwent seizure attacks of right hand and leg in the night and lasted for 3 minutes each time. Head MRI showed left midcingulate lesion. (Figure 4.) DTI showed the pyramidal tract was located on the lateral side of the tumor. The aslant frontal tract mainly passed through the anterolateral side of the tumor. In order to protect the primary motor area and subcortical tracts, surgical approach was transcortical approach incising superior frontal gyrus on medial side. The surgical plan was to resect tumor under intraoperative awake anesthesia and IONM (Intraoperative Neurophysiological Monitoring). During the operation, the incision point of medial frontal cortex was determined with motor evoked potential monitoring. During resecting period, subcortical electrical stimulation was used to confirm the distance to pyramidal tract and delineate "safe zone". Histopathology confirmed diagnosis of “anaplastic astrocytoma, WHO grade III, IDH mutant type”. Regarding function outcome, arrest of speech and hemiparesis of leg within postoperative short-term, but finally motor and language function recovered to normal when six-month follow-up.

Illustrative case 3

The patient was a 70-year-old male. The patient experienced weakness of his left limbs, disorder in motivation control, decreasing physical activity, and difficulty in alternating movements for two months. Head MRI revealed right posterior cingulate cortex lesion, high-grade glioma possible. (Figure 5.) DTI showed that tumor was mainly located posterior to Pyramidal Tract on medial side. The right cingulum bundle mainly passed through both sides of the tumor. For protection of motor function, intraoperative cortical motor evoked potential (MEP) real-time monitoring combined with subcortical electrical stimulation technology would be used. It was difficult to pull the medial side of the cerebral hemisphere outwards because damage might be caused to the briging veins. In this case trans precuneus cortical approach was chosen. The surgical plan was to resect tumor under intravenous anesthesia. Histopathology confirmed diagnosis of “Glioblastoma, WHO grade IV. IDH wildtype. Adjuvant radio-therapy and chemo-therapy were carried out. The chemo-therapy was advised temozolomide with Stupp regimen by neuro-oncologist. Until six-month postoperative follow-up, MRI scanning showed tumor no progression and patient complained no morbidity of limbs except memory deficits.


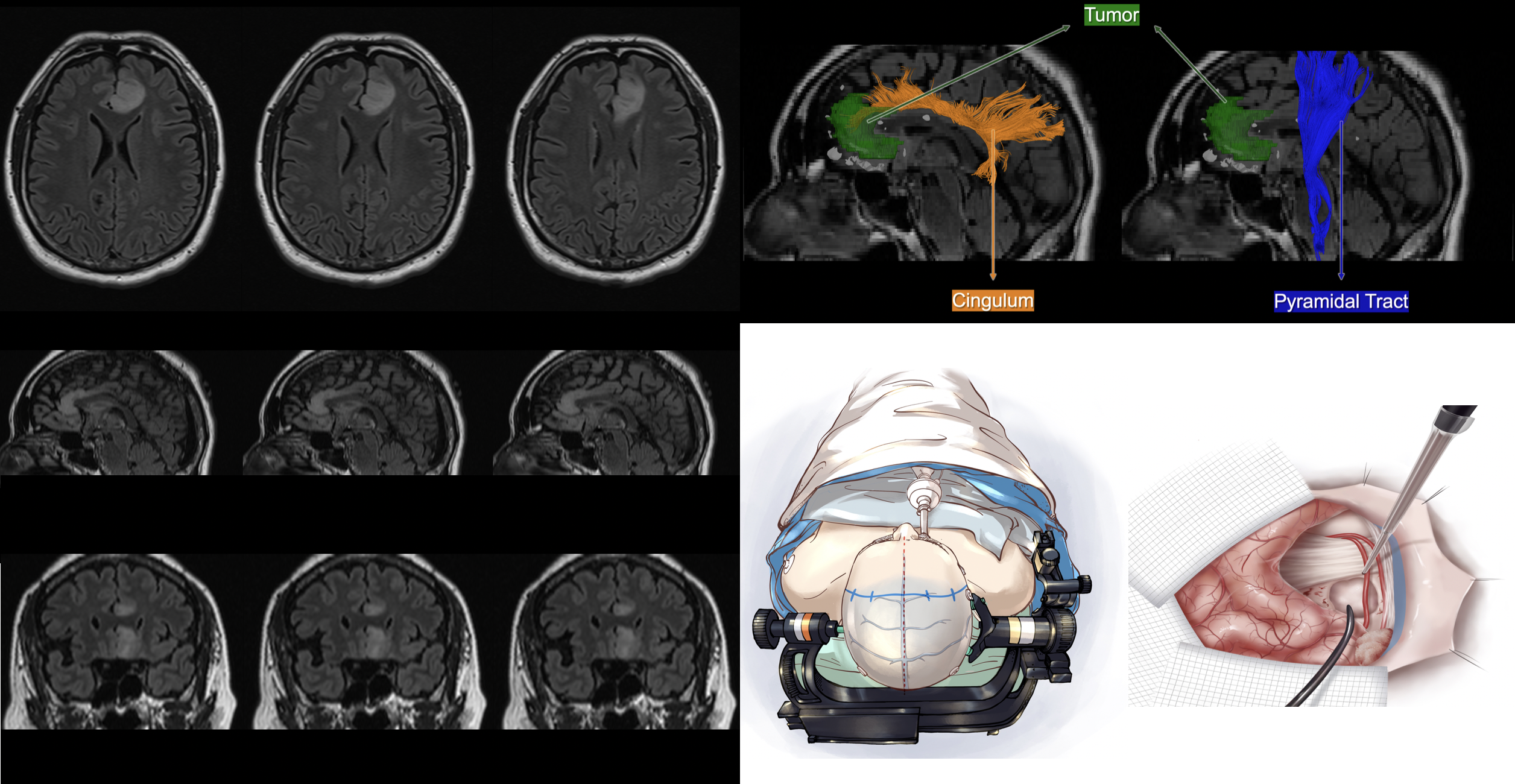


Figure 3. The MRI imaging, multi-modality fusion navigation, surgical position and approach of pure anterior cingulate glioma


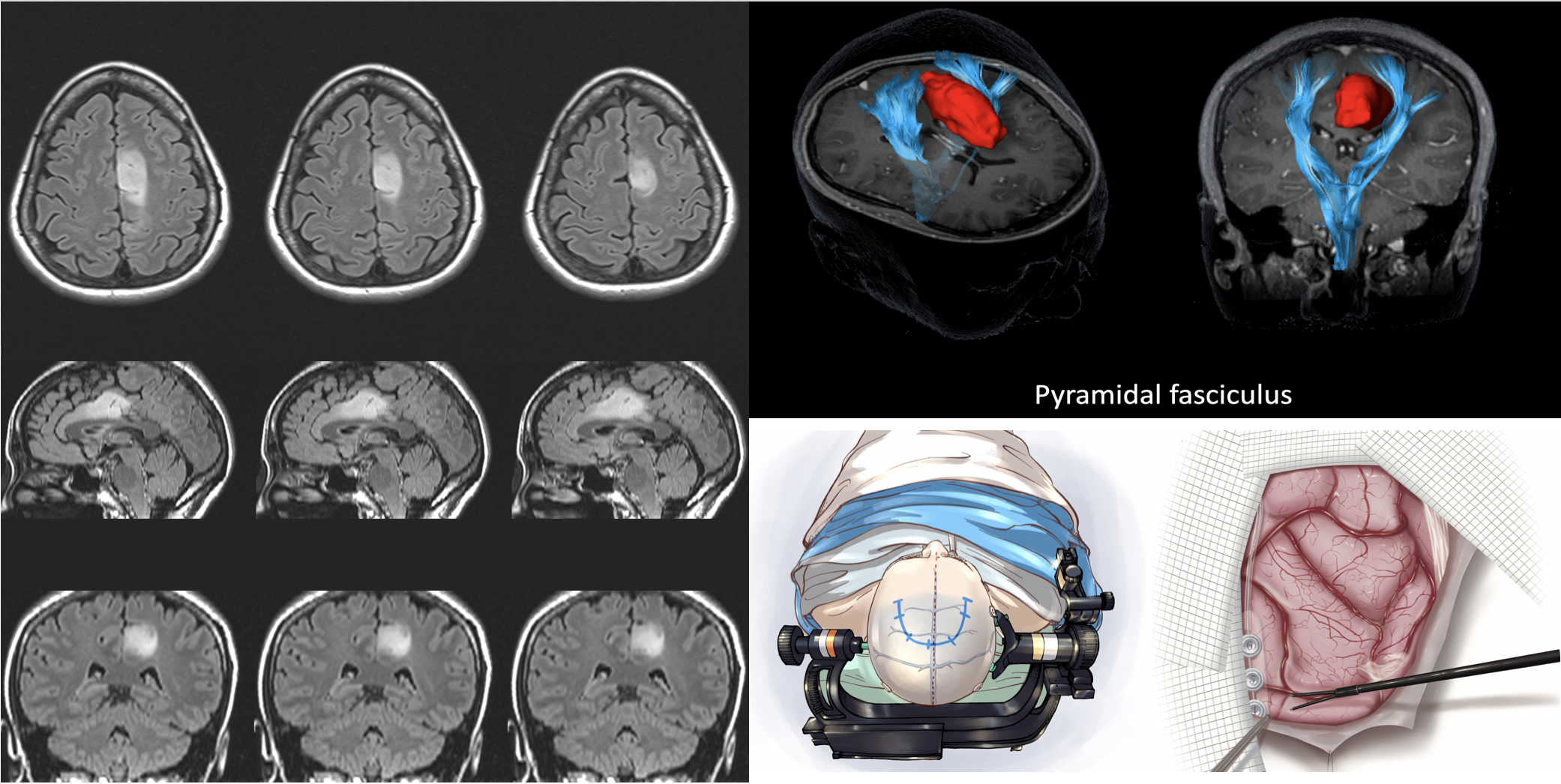


Figure 4. The MRI imaging, multi-modality fusion navigation, surgical position and intraoperative neurophysiological monitoring of pure midcingulate glioma
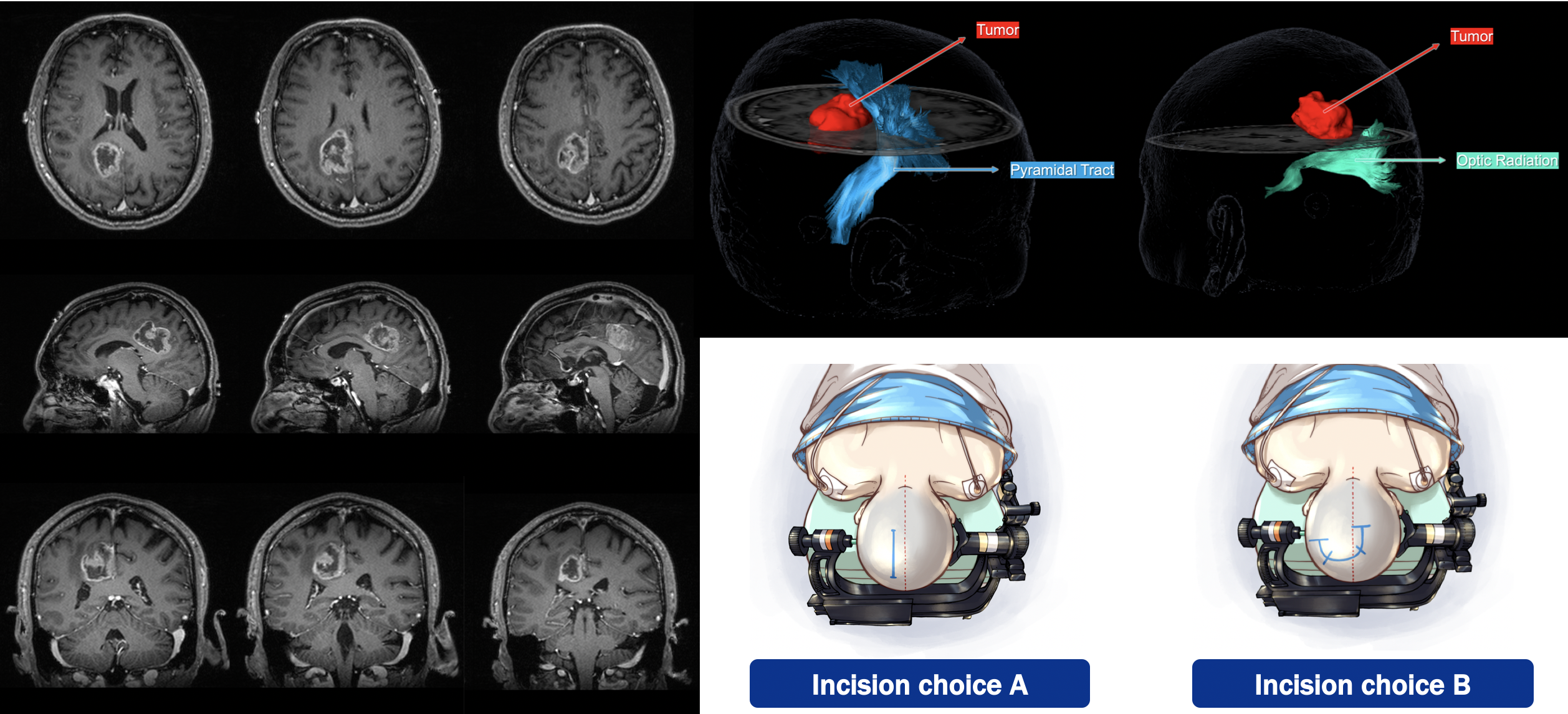


Figure 5. The MRI imaging, multi-modality fusion navigation, surgical position and selective incision of pure posterior cingulate glioma (Incision choice B actually used in the case)
